# The functions and clinical significance of circRNAs in hematological malignancies

**DOI:** 10.1186/s13045-020-00976-1

**Published:** 2020-10-17

**Authors:** Xiangxiang Zhou, Linquan Zhan, Kai Huang, Xin Wang

**Affiliations:** 1 Department of Hematology, Shandong Provincial Hospital, Cheeloo College of Medicine, Shandong University, Jinan, 250021 Shandong People’s Republic of China; 2 Department of Hematology, Shandong Provincial Hospital Affiliated to Shandong First Medical University, Jinan, 250021 Shandong People’s Republic of China; 3 School of Medicine, Shandong University, Jinan, 250012 Shandong People’s Republic of China; 4 Shandong Provincial Engineering Research Center of Lymphoma, Jinan, 250021 Shandong People’s Republic of China; 5 Branch of National Clinical Research Center for Hematologic Diseases, Jinan, 250021 Shandong People’s Republic of China; 6 National Clinical Research Center for Hematologic Diseases, The First Affiliated Hospital of Soochow University, Suzhou, 251006 People’s Republic of China; 7 Department of Chemotherapy, Cancer Center, Qilu Hospital of Shandong University, Jinan, 250012 Shandong People’s Republic of China

**Keywords:** Circular RNAs, Hematological malignancies, Tumorigenesis, Drug-resistance, Biomarker

## Abstract

With covalently closed circular structures, circular RNAs (circRNAs) were once misinterpreted as by-products of mRNA splicing. Being abundant, stable, highly conserved, and tissue-specific, circRNAs are recently identified as a type of regulatory RNAs. CircRNAs bind to certain miRNAs or proteins to participate in gene transcription and translation. Emerging evidence has indicated that the dysregulation of circRNAs is closely linked to the tumorigenesis and treatment response of hematological malignancies. CircRNAs play critical roles in various biological processes, including tumorigenesis, drug resistance, tumor metabolism, autophagy, pyroptosis, and ferroptosis. The N6-methyladenosine modification of circRNAs and discovery of fusion-circRNAs provide novel insights into the functions of circRNAs. Targeting circRNAs in hematological malignancies will be an attractive treatment strategy. In this review, we systematically summarize recent advances toward the novel functions and molecular mechanisms of circRNAs in hematological malignancies, and highlight the potential clinical applications of circRNAs as novel biomarkers and therapeutic targets for future exploration.

## Background

**Fig. 1:**
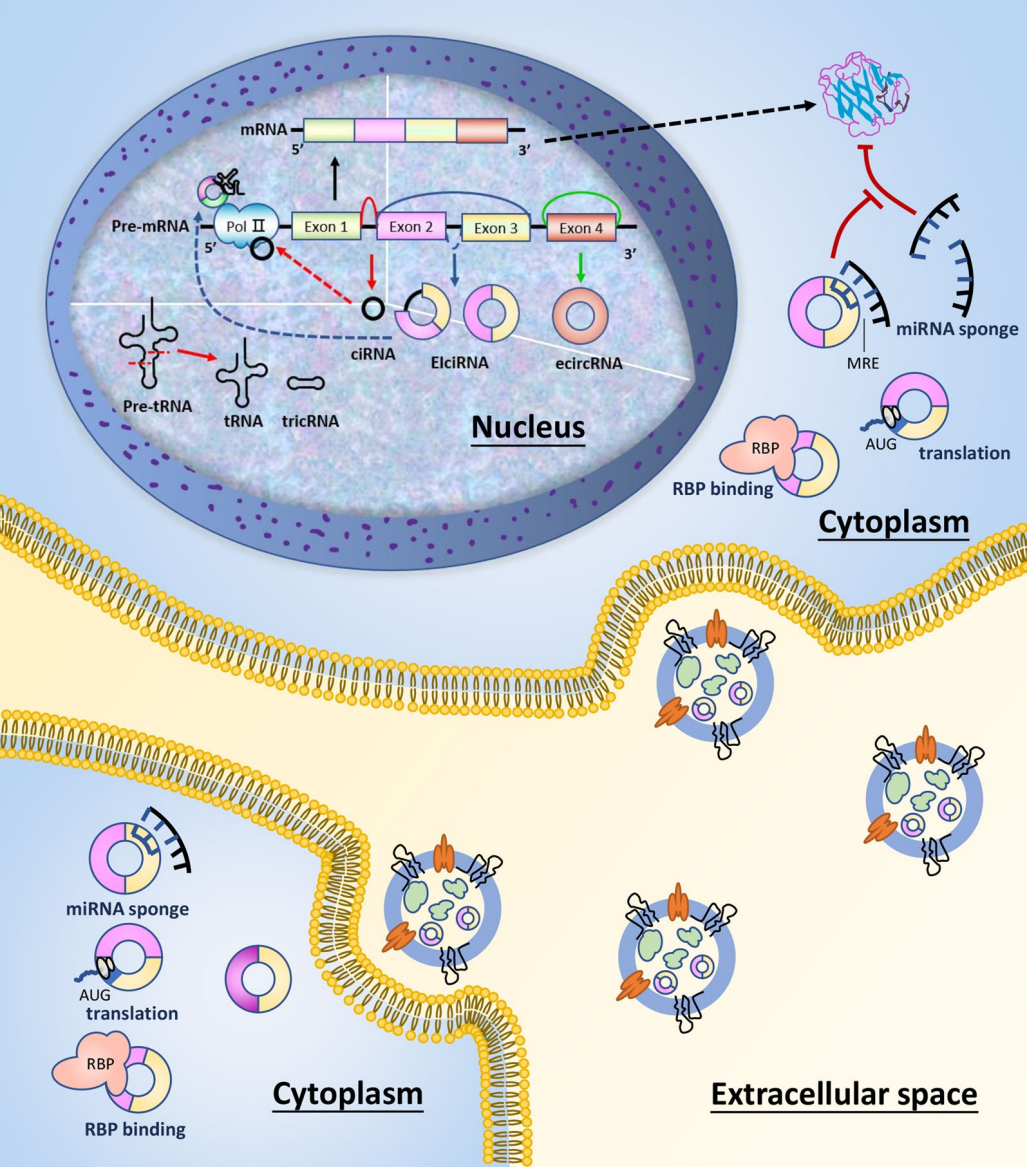
The biogenesis and function of circRNAs. CircRNAs are classified into 4 types: ecircRNAs, ciRNAs, EIciRNAs, and tricRNAs. CircRNAs function as regulators of transcription and translation, which includes miRNA binding, protein binding, gene expression regulating, and acting as templates for translation

Circular RNAs (circRNAs) are a novel type of competing endogenous RNAs (ceRNAs) of the non-coding RNA (ncRNA) families. Without 5′ and 3′ ends, they are hallmarked by covalently closed continuous loops and are more stable than linear RNAs in vivo [1]. CircRNAs are abundant in biological cells, highly conserved, and expressed in a tissue-specific pattern [2]. CircRNAs are classified into four categories, including exon circRNAs (ecRNAs), circular intron RNAs (ciRNAs), exon-intron circRNAs (EIciRNAs), and tRNA intronic circular RNAs (tricRNAs) (Fig. 1). Accumulating evidence has revealed various biological functions of circRNAs, which have attracted widespread attention [3]. Localized in miRNA binding sites, circRNAs could directly sponge miRNAs through miRNA response elements (MREs), thereby negatively regulating the inhibition of target mRNAs [4]. CircRNAs also regulate gene expression and bind to RNA-binding proteins (RBPs), playing critical roles in gene transcription and translation [5, 6]. In addition, the translation potential of circRNAs as protein templates related to tumorigenesis and development has been proved [7]. Since circRNAs are abundant in human peripheral blood and tissues, making them easy to detect [8, 9]. CircRNAs have been illuminated to participate in various biological and physiological processes, containing cell growth, metastasis, stemness, tumor microenvironment, and immune evasion [10, 11], suggesting potential contributions to the pathogenesis of several human diseases.

**Fig. 2:**
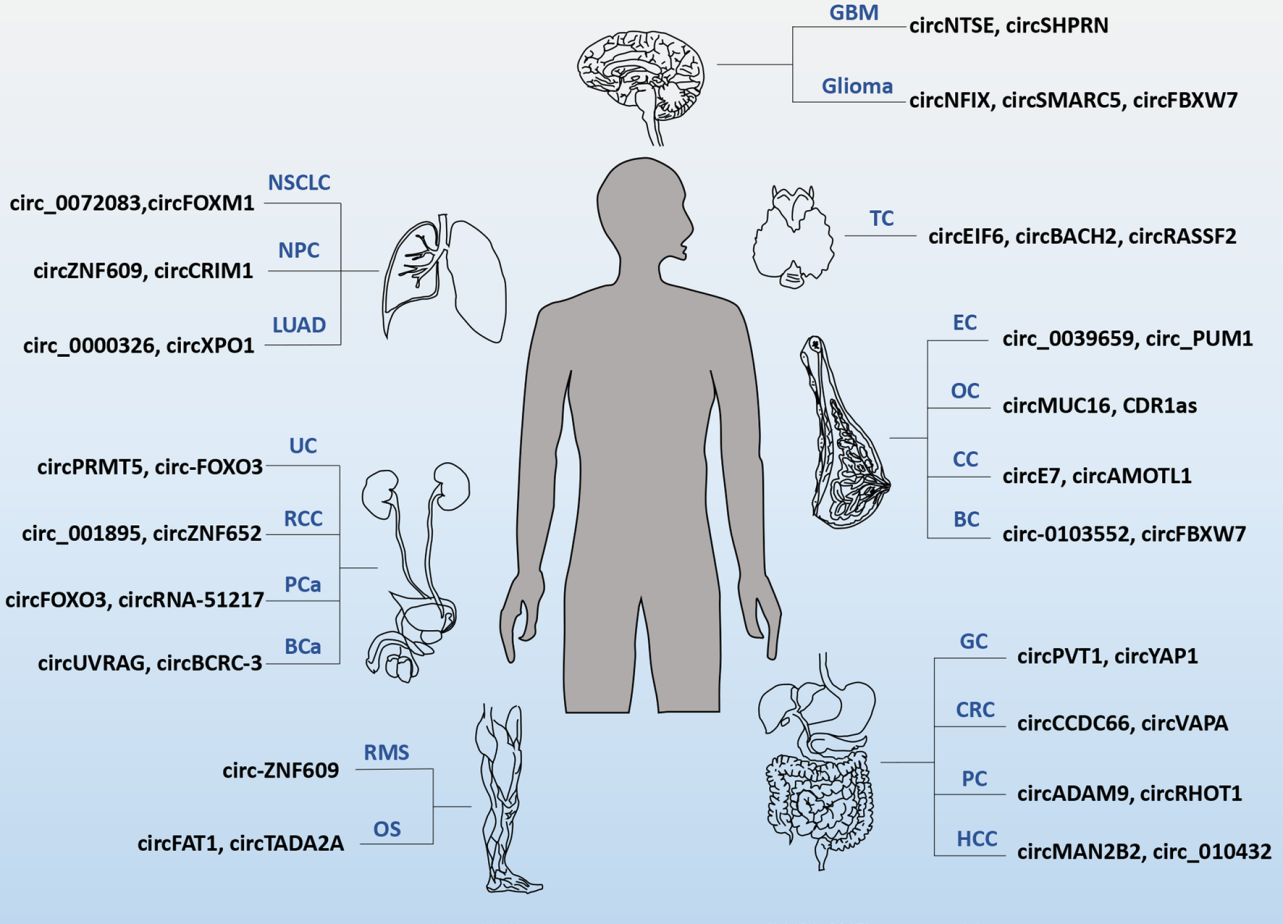
CircRNAs differentially expressed in cancers. GC: gastric cancer; CRC: colorectal cancer; PC: pancreatic cancer; HCC: hepatocellular carcinoma; BC: breast cancer; CC: cervical cancer; OC: ovarian cancer; EC: endometrial cancer; NSCLC: non-small cell lung cancer; LUAD: lung adenocarcinoma; NPC: nasopharyngeal carcinoma; BCa: bladder cancer; PCa: prostate cancer; RCC: renal cell carcinoma; UC: urothelial carcinoma; GBM: glioblastoma; TC: thyroid carcinoma; OS: osteosarcoma; RMS: rhabdomyosarcoma

CircRNAs act as tumor suppressors or oncogenes to participate in the development of a variety of tumors and are becoming novel diagnostic and prognostic biomarkers [12]. The differential expression and function of circRNAs in a variety of cancers have been identified (Fig. 2) [13]. Recently, emerging evidence suggests that circRNAs play vital roles in the tumorigenesis and progression of hematological tumors [14, 15]. Moreover, circRNAs are affective in iron metabolism and N6-methyladenosine (m^6^A) modification [16, 17]. The artificial circRNAs molecules targeting miRNAs and nanoparticle-based delivery systems provide novel therapeutic prospects [18]. Given that emerging literature has summarized the expression patterns and classical functions of circRNAs in hematological malignancies, here, we focus on the current state of knowledge regarding the novel mechanisms and potential clinical applications of circRNAs among hematological tumors.

## Functions of circRNAs in hematological malignancies

### Facilitating tumorigenesis of hematological malignancies

Through genome-wide studies of myeloid leukemia, aberrantly expressed circRNAs in acute myeloid leukemia (AML) and chronic myeloid leukemia (CML) were identified [19, 20]. A total of 569 differentially expression circRNAs (DECs) were screened by circRNA microarray in 6 bone marrow samples from pediatric AML patients, with 273 circRNAs upregulated and 296 downregulated. Functional investigation illustrated that circ-0004136 promoted the proliferation of AML cells by sponging miR-142 [21]. Moreover, circRNA-sequencing of bone marrow samples from AML patients identified the differentially expression of circ_0009910, which promoted the growth of AML cells by downregulating miR-20a-5p [19]. Han et al*.* found that circ_0001947 curbed cell proliferation by targeting the miR-329-5p/CREBRF axis in AML [22]. Circ_100290, highly expressed in AML, promoted proliferation and restrained apoptosis of AML cells by sponging miR-203 to regulate Rab10 expression [23].

In addition, recent studies have demonstrated that circRNAs could also modulate cell proliferation independent of their related linear RNAs [3]. Circ_0121582, a product of the reverse splicing of GSK3β exon 1 to exon 7, was confirmed to suppress the growth of AML cells [24]. Interestingly, different mechanisms were regulated by circ_0121582 in the cytoplasm and nucleus, respectively. Circ_0121582 formed a sponge with the miR-224/GSK3β axis in the cytoplasm, and bound to the promoter of GSK3β to recruit TET1 in the nucleus [24]. The occurrence of internal tandem duplication (ITD) mutations in the juxtamembrane domain of the FMS-like tyrosine kinase-3 (FLT3) gene (FLT3-ITD) is identified in up to 30% of AML patients, suggesting a significantly worse clinical outcome. Sun et al*.* reported that circMYBL2 promoted the proliferation of FLT3-ITD-positive AML cells by directly interacting with the RBP PTBP1 in vitro and in vivo [25]. Moreover, knocking down circMYBL2 decreased the phosphorylation of FLT3 kinase in ITD mutant cells, and further weakened the phosphorylation of STAT5, the downstream target of FLT3 critical for AML progression [25]. Another circRNA recently identified in FLT3-ITD-positive AML was circ_0000370, derived from the FLI-1 gene, which was associated with FLT3-ITD. Circ_0000370 facilitated the viability and suppressed apoptosis of FLT3-ITD-positive AML cells by modulation of miR-1299 and S100A7A [26].

In terms of lymphocytic leukemia, knockdown of circPVT1 accelerated the apoptosis of acute lymphocytic leukemia (ALL) cells by declining the expression of c-Myc and Bcl-2 [27]. Upregulation of circ-0000745 resulted in enhanced proliferation of ALL cells by activating ERK [28]. Transcriptomic sequencing of 21 de novo chronic lymphocytic leukemia (CLL) patients revealed differentially expression of 859 circRNAs distinguished CLL cells from normal B cells [29]. It was recently demonstrated that circ_0132266 participated in CLL tumorigenesis through interacting with miR-337-3p to modulate PML expression [30]. Xia and colleagues demonstrated that circ-CBFB contributed to CLL progression by modulation of the miR-607/FZD3/Wnt/β-catenin cascade [31].

**Fig. 3:**
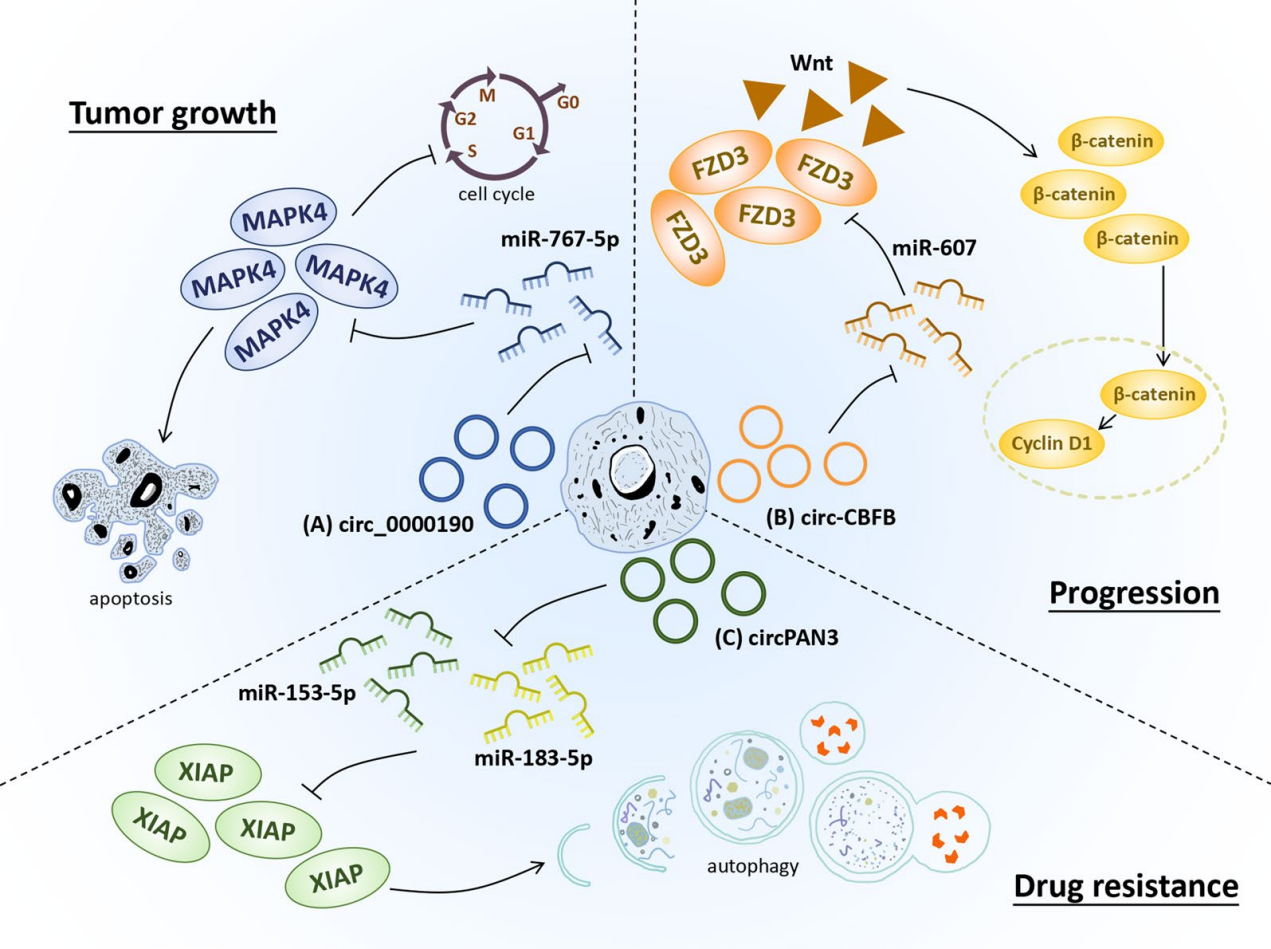
CircRNA-miRNA-mRNA networks in hematological malignancies. Selected samples of circRNAs and their genomic targets are exhibited for tumor growth, progression and drug resistance. (A) Circ_0000190 was downregulated in MM and inhibited proliferation as well as induced apoptosis of MM cells through negatively regulating the suppression of miR-767-5p to MAPK4, which led to tumor growth. (B) Circ-CBFB, overexpressed in CLL, was identified as a sponge of miR-607 that targeted FZD3. Circ-CBFB promoted FZD3 expression, resulting in activation of the Wnt/β-catenin pathway and consequent progression of CLL. (C) CircPAN3 could inhibit both miR-153-5p and miR-183-5p, thereby upregulating the expression of XIAP. CircPAN3 was also responsible for AML drug resistance via regulating the level of autophagy-associated proteins

Currently, studies of circRNAs in lymphoma are relative rare, with only some subtypes reported. Ectopic expression of circ-LAMP1 was detected in T-cell lymphoblastic lymphoma (T-LBL) tissues, which promoted T-LBL progression by sponging miR-615-5p and activating DDR2 level [32]. Augmented expression of circ-APC inhibited the growth of diffuse large B-cell lymphoma (DLBCL) cells in vitro and in vivo by recruiting TET1 to the promoter of APC, subsequently resulted in the activation of canonical Wnt/β-catenin signaling pathway [33]. Remarkable overexpression of circ-CDYL was detected in the plasma of mantle cell lymphoma (MCL) and multiple myeloma (MM) patients [34, 35]. Knockdown of circCDYL could inhibit DNA synthesis rate and cell activities to stunt MM progression. Silencing circCDYL decreased the expression of YAP, the key effector of Hippo signaling, and upregulated miR-1180 in MM xenograft model [35, 36]. Circ_0000190, downregulated in the tissues and plasma of MM patients, restrained the proliferation of MM cells through modulating the miR-767-5p/MAPK4 axis [37]. The overexpressed circ_0000142 in MM targeted miR-610 to promote the level of AKT3 mRNA and enhance cell growth, migration and invasion [38]. Moreover, circ_0007841 directly interacted with miR-338-3p to accelerate MM progression [39]. All these results provide evidence that circRNAs mainly function as sponges of miRNAs and form circRNA/miRNA/mRNA axes to participate in the tumorigenesis of hematological cancers (Fig. 3). Nevertheless, further investigations are still needed to explore the detailed mechanism and potential clinical application.

### Participating in drug resistance

With the clinical application of novel anti-tumor drugs, the efficacy of hematological tumors has been remarkably improved. However, drug resistance is still a bottleneck that hinders better prognosis of patients. CircRNAs are proved to perform pivotal parts in chemoresistance by reducing drug concentration, activating downstream signaling pathways, and modulating DNA repair ability [40, 41]. Li et al*.* constructed a doxorubicin (ADM)-resistant cell line (HL-60/ADM) and screened differentially expressed ncRNAs using high-throughput sequencing, 1824 circRNAs included. Targets of DECs were enriched in ribonucleoside and purine ribonucleoside triphosphate catabolic process, intracellular, adenyl ribonucleotide binding, and several classical signaling pathways through Gene Ontology (GO) and Kyoto Encyclopedia of Genes and Genomes (KEGG) enrichment analyses, indicating that circRNAs may participate in drug resistance in AML via modulating multiple biological processes [42]. Moreover, circPAN3 was validated to mediate ADM-resistance of AML cells via targeting the miR-153-5p/miR-183-5p/XIAP axis as well as enhancing autophagy activity, and to promote the expression of apoptosis-marked proteins [14, 43]. Knockdown of circMYBL2 accelerated apoptosis and inhibited proliferation of AML cells resistant to quizartinib, a selective and efficient FLT3 inhibitor, indicating circMYBL2 inducing quizartinib resistance in AML [25].

The application of tyrosine kinase inhibitors (TKIs), represented by imatinib (IM), has doubled the 5-year overall survival (OS) of CML patients [44]. Nevertheless, TKI resistance is gradually being common in clinical practice. Several studies have demonstrated the participation of circRNAs in drug resistance of CML cells. Knocking down circ_0080145 in IM-resistant CML cell lines (K562/IM, KU812/IM) suppressed the glycolysis process and cell growth and induced apoptosis through circ_0080145/miR-326/PPFIA1 axis [15]. Besides, the expression of circBA9.3 was positively related to the BCR-ABL level among TKI-resistant CML patients. Mechanistically, circBA9.3 strengthened the antiapoptotic properties of K562 cells and upregulated the levels of both ABL1 and BCR-ABL1 proteins to reduce the sensitivity of leukemia cells to TKIs, thereby promoting resistance against TKI therapy [45]. Circ_100053 was overexpressed in IM-resistant CML patients and K562/IM cells and was associated with advanced clinical stages and the mutation status of BCR-ABL, indicating the contribution to IM resistance of CML, nevertheless, with the unclear mechanism [46]. As a miR-34a-5p sponge, circ_0009910 could promote cell proliferation, block apoptosis in K562/R cells, and activate ULK1-induced autophagy, leading to increased resistance of CML cells to IM [47].

As sponges of different miRNAs, circRNAs may participate in different mechanisms to induce drug resistance in the same disease. Circ_0007841 was involved in the bortezomib (BTZ) tolerance [48] and ADM-resistance in MM cells, and induced chemoresistance by activating ABCG2 [49]. Taken together, circRNAs mediate chemoresistance by regulating signaling transduction pathways or forming ceRNA regulatory networks, and may act as novel therapeutic targets to improve the efficacy of refractory/relapsed patients.

### Regulation of tumor metabolism

Represented by glycolysis, fatty acid oxidation, and amino acid metabolism, metabolic reprogramming is a typical hallmark in cancer cells [50]. Tumor cells are featured by the strong dependence on glycolysis to provide energy despite sufficient oxygen availability [51]. Targeting glycolysis has been revealed to be a promising therapeutic strategy for hematological malignancies [52]. Hexokinase-2 (HK2) and glucose transporters (GLUT) act as critical modulators of glycolysis progress [53]. A recent study confirmed that silencing circ_0080145 suppressed the glucose consumption, lactic acid content, and the HK2 levels in IM-resistant CML cell lines, indicating the regulatory effect of circ_0080145 on glycolysis in IM-resistance [15]. Aberrant high expression of circ_100290 upregulated GLUT1 by targeting miR-378a to promote the glycolysis in oral squamous cell carcinoma (OSCC) cells [54]. Circ_100290 was also overexpressed in AML, co-expressed with Rab10 [23]. As a target of miR-432-5p, Rab10 participated in the decreased glycolysis induced by miR-432-5p in glioma [55]. Knockdown of circ-PVT1 reduced the glycolytic metabolism of OSCC cells by targeting the miR-106a-5p/HK2 axis [56]. Although circ-PVT1 has been found to be involved in the occurrence of ALL, its regulation on the glucose metabolism of hematological malignancies has not yet been reported. In addition, further investigations on the specific function of circRNAs and glycolysis in hematological tumors are warranted.

Increased de novo synthesis of fatty acids and fat accumulation present in diverse tumors are responsible for tumor development [57]. The content of lipoproteins is tightly connected with the phenotypic and clinical characteristics of hematological malignancies [58]. Recent studies have clarified that circRNAs maintained the global adipocyte transcriptional program of lipid biosynthesis and metabolism [59]. Moreover, circFASN enhanced the promotion of tacrolimus on triglyceride accumulation [60]. Nevertheless, the mechanism of circRNAs in lipid metabolism of malignancies is still unclear, which is expected to become a novel field in the study of circRNAs in hematological tumors.

### Novel functions of circRNAs

#### Inducing autophagy

Autophagy is a primary intracellular degradation process regulating tumorigenesis and associated with the sensitivity of tumor cells to chemotherapeutic drugs [61]. Emerging discoveries indicated that circRNAs played significant roles in tumor autophagy [62]. Enhanced autophagic activity was found in ADM-resistant AML cell lines, THP1/ADM and K562/ADM. Silencing circPAN3 reduced the levels of autophagy markers, including the LC3-II/LC3I ratio and Beclin-1. The targeted miRNAs of circPAN3, analyzed by target prediction database, were related to AMPK signaling pathway and downregulated in THP-1 cells with circPAN3 overexpression. Taken together, circPAN3 may activate MAPK pathway to enhance autophagy to induce chemo-resistance in AML [14].

Silencing circ_0009910 resulted in downregulation of ULK1, an autophagy promoter overexpressed in IM-resistant K562 cells. It was further confirmed that circ_0009910 could activate ULK1-induced autophagy via sponging miR-34a-5p, thereby promoting the IM resistance of CML cells [47]. CircCDYL was demonstrated to enhance the autophagic level in breast cancer through miR-1275/ATG7/ULK1 axis [63]. It is worth noting that upregulated levels of circCDYL were also detected in MCL and MM and might serve as potential biomarkers for diagnosis [34, 35]. However, the involvement of circCDYL in the autophagy of hematological malignancies still needs further investigation. Although investigations on circRNAs in tumor autophagy are still in infancy, it will bring new opportunities for future diagnosis and treatment of hematological malignancies.

#### Regulating pyroptosis

Pyroptosis is an inflammasome-activated programmed cell death pathway, characterized by the immediate formation of pores in cell membrane and increased permeability [64]. Induction of pyroptosis represents a novel potential therapeutic strategy for hematological malignancies [65]. The latest studies have illuminated the regulation of circRNAs on pyroptosis in human diseases. CircACTR2, upregulated in high-glucose-treated HK-2 cells, was proved to increase pyroptosis by evaluating propidium iodide (PI) uptake and lactate dehydrogenase (LDH) level [66]. Besides, circ_0076631, overexpressed in glucose-stressed cardiomyocytes and serum of diabetic patients, was validated to activate pyroptosis via circ_0076631/miR-214-3p/caspase-1 axis in diabetic cardiomyopathy [67]. High-throughput sequencing analysis showed the high expression of miR-214-3p in primary cutaneous follicle center lymphoma (PCFCL), suggesting the potential of circRNA/miRNA axis in pyroptosis of lymphoma [68]. The participation of circHIPK3 in pyroptosis was recently reported. CircHIPK3 could downregulate miR-421, leading to the increased FOXO3a expression, thereby inhibiting pyroptosis and releasing IL-1β and IL-18 [69]. Previous studies have found that the high expression of circHIPK3 in CML was significantly associated with poor prognosis [70]. Besides, AKT/FOXO3a pathway was involved in the apoptosis of AML [71]. Genetic polymorphisms of IL-18 and IL-1β were confirmed related to the prognosis of AML [72]. However, whether circHIPK3 could influence the pyroptosis level in leukemia remains further investigations. Overall, the regulation of circRNAs on pyroptosis is a hopeful therapeutic target, but its role in hematological malignancies is yet to be fully understood.

#### Function of circRNAs in ferroptosis

Different from autophagy and apoptosis, ferroptosis is an iron- and reactive oxygen species (ROS)-dependent form of programmed cell death activated by iron oxidation [73, 74]. Accumulating studies have reported the regulatory mechanisms of ncRNAs in ferroptosis of tumors, but few of them focused on circRNAs [75]. Xu et al*.* reported that circIL4R accelerated tumorigenesis and refrained ferroptosis by regulating the miR-541-3p/GPX4 axis in HCC [76]. Circ-TTBK2 knockdown delayed proliferation and invasion, as well as promoted ferroptosis of glioma cells by regulating the miR-761/ITGB8 axis [77]. GPX4, a key regulator of ferroptosis, was overexpressed in primary MM cells [78]. However, both the circRNA/miRNA/GPX4 axis and its function in ferroptosis in MM have not been revealed. Although the relationship between circRNAs and ferroptosis in hematological malignancies has not been reported before, emerging evidence suggests that the changes in iron metabolism are central characters of leukemia [79]. Typhaneoside (TYP) was proved to prevent AML progression through triggering autophagy and ferroptosis. Therefore, targeting iron metabolism, such as ferroptosis, is likely to provide promising therapeutic options for individualized treatment of leukemia.

#### circRNAs and m^6^A modification

m^6^A methylation has emerged recently as the novel mechanism of RNA modification, which executes important functions in malignant hematopoiesis, including AML [80]. The interplay between m^6^A modification and circRNAs provides novel insights into the therapeutic strategy of malignancies [81, 82]. Zhou et al. demonstrated that the written and read complexes of m^6^A modification in circRNAs were the same as those of mRNAs, but the modification patterns were distinct [83]. A growing body of evidence indicates that m^6^A modification could modulate the production and function of circRNAs. The m^6^A circRNAs expressed in a cell-type-specific pattern, indicating that m^6^A modification of circRNAs may exert different biological functions in different cell types [83, 84].

m^6^A modification of circRNA inhibits innate immunity by blocking RIG-I activation. Moreover, the m^6^A reader YTHDF2 could bind to m^6^A-circRNA, thereby suppressing the innate immunity [17]. Chen et al*.* identified that circNSUN2 could form a circNSUN2/IGF2BP2/HMGA2 RNA-protein ternary complex to enhance the stability of HMGA2 mRNA in colorectal carcinoma [85]. Microarray analysis of DECs in poorly differentiated gastric adenocarcinoma revealed that most DECs had m^6^A modification, and the trend of m^6^A modification changes was consistent with the expression level of circRNAs [86]. Circ_0001105 suppressed the progression of osteosarcoma through sponging miR-766 to enhance the expression of YTHDF2 [87]. Interestingly, the inhibition of YTHDF2 selectively targeted leukemic stem cells (LSCs) in AML, indicating the potential functions of circRNA regulated YTHDF2 in the progression of AML [88]. In addition, circ_KIAA1429 could promote the progression of HCC by regulating the m^6^A-YTHDF3-Zeb1 [89]. It was confirmed that SNHG14/miR-5590-3p/Zeb1 axis enhanced the progression and immune evasion through regulating PD-1/PD-L1 checkpoint in DLBCL, which provide an innovative perspective for circRNA mediated immunotherapy of lymphoma [90]. Nevertheless, the m^6^A modification of circRNAs in hematological malignancies has rarely been reported yet. Further studies on how m^6^A modification modulates the production and function of circRNAs in hematological malignancies will improve our understating of the biological function of circRNAs.

#### Function of fusion circRNAs

Gene fusion is a central class of somatic mutational events in hematological malignancies through chromosomal rearrangements triggered by DNA double-strand breaks. As high-risk factors of AML, cytogenetic abnormalities are featured by fusion proteins, including AML1-ETO, PML-RARα, and MLL/AF9, originated from chromosomal translocations, which have been acknowledged as specific biomarkers for prognosis [91]. Additionally, translocations produce not only fusion mRNAs but also fusion circRNAs (f-circRNAs) [92]. Despite the lack of specific mechanisms, f-circRNAs are oncogenic in in vitro and in vivo models [93]. Human mixed lineage leukemia (MLL) gene is involved in chromosome translocations with a multitude of partners, such as AF9 (MLLT3) [94]. Several f-circRNAs have been identified from MLL fusion genes, including MLL-AF9, MLL-AF4, and MLL-ENL [95]. The MLL-AF9 fusion gene is predominantly expressed in AML. Sanger sequencing revealed the existence of f-circM9_1 and f-circM9_2 in THP1 cells. F-circM9 overexpression suppressed apoptosis induced by cytarabine (Ara-C) and arsenic trioxide (ATO) in K562 cells. Moreover, the spleens of mice transplanted with f-circM9 overexpressed leukemia cells were relatively bigger with more leukemia cells, and decreased apoptosis in bone marrow when treated with Ara-C [93]. AF4 is another partner of MLL fusion genes. Four circRNAs were detected from AF4 gene, including circAF4 (EX3-4), circAF4 (EX3-5), circAF4 (EX5-6), and circAF4 (EX12). CircAF4 was upregulated in leukemia patients and cells with MLL-AF4 translocation. CircAF4 enhanced proliferation and blocked apoptosis through circAF4/miR-128-3p/MLL-AF4 axis. Knockdown of circAF4 extended survival times of mice [96].

## Potential clinical application of circRNAs

### Promising biomarkers for diagnosis and prognosis

CircRNAs are widely and conservatively expressed in hematopoietic cells [97]. As a result of their abundancy and accessibility, circRNAs are expected to be ideal biomarkers in the diagnosis and prognosis of hematological malignancies. Among all the circRNAs, PVT1 has been considered to participate in the pathogenesis of hematological malignancies [98, 99]. CircPVT1 showed increased expression in ALL, pediatric B-precursor ALL and AML cases harboring MYC amplifications in the form of dmin, hsr, or ring chromosomes (AML-Amp) [27, 100, 101]. Silencing circPVT1 was validated to inhibit cell proliferation and induce apoptosis in ALL [27]. Additionally, circPVT1 was overexpressed in AML-Amp cases leading to the identification between various karyotypes of AML [101].

Due to the high heterogeneity of hematological malignancies and the cell-type specificity of circRNAs, there are specific expressions of circRNA in different types of hematological diseases (Table 1). At present, a variety of circRNAs have performed promising function for evaluating prognostic model, such as circ_0003602, circ_0005571, circ_0074371, circ_0007609, circ_0012152, hsa_circ_0001857 and circ_0001247 [102, 103]. Circ-Foxo3, Circ-RPS6KB1, circ-CSMD2, circ-ANXA2, circ-PWP2, circ-RBM5, circ-ZZEF1, circ-GSK3B and circ-FOXP1 could potentially identify AML patients from healthy groups [104, 105]. Among them, circ-ANXA2 overexpression was related to shorter event-free survival (EFS) and OS of AML patients. Meanwhile, AML patients achieved complete remission (CR) presented lower level of circ-ANAX2 than those did not reach CR, accompanied by longer EFS and OS [105]. Receiver operating characteristic (ROC) curve analysis revealed that the expression of circ-VIM could distinguish AML patients from healthy groups. Highly expressed circ-VIM acted as an independent prognostic factor for OS and leukemia-free survival (LFS) in AML [106]. Interestingly, the expression level of the same circRNA differs in subtypes of the same disease, highlighting the specificity of circRNAs as biomarkers. Circ_0075001 was overexpressed in M0 or M1 subtype of AML patients and significantly downregulated in M2, M4 and M5 subgroups, showing the potential to distinguish the differentiation degree of the AML [107].

**Table 1:** Circular RNAs implicated in hematological malignancies

Disease	CircRNA	Expression	Phenotype	Clinical significance	Possible target/mechanism	Ref.
AML	circRNA-DLEU2	Up	proliferation ( +), apoptosis (−), tumor formation ( +)	/	miR-496/PRKACB	[125]
	circ_100290	Up	proliferation ( +), apoptosis (−)	/	miR-203/Rab10	[23]
	circPAN3	Up	autophagy ( +), apoptosis (−), ADM-resistance ( +)	/	miR-153-5p/miR-183-5p/XIAP	[14, 43]
	circ-ANXA2	Up	proliferation ( +), apoptosis (−)	high disease risk, poor risk stratification, low CR level, short EFS and OS	miR-23a-5p/miR-503-3p	[105]
	circ_0009910	Up	proliferation ( +), apoptosis (−)	poor risk, short OS	miR-20a-5p	[19]
	circ_0000370	Up	proliferation ( +), apoptosis (−), cell cycle ( +)	FLT3-ITD +	miR-1299/S100A7A	[26]
	circMYBL2	Up	proliferation ( +), quizartinib resistance ( +)	FLT3-ITD +	PTBP1, FLT3 kinase translational ( +)	[25]
	circ-VIM	Up	/	distribution of WBC count, FAB subtypes, short OS and LFS	/	[106]
	circ-0004136	Up	proliferation ( +)	/	miR-142	[21]
	circ-HIPK2	Down	differentiation ( +)	ATRA-induced differentiation	miR-124-3p	[111]
	circ_0001947	Down	proliferation (−), apoptosis ( +)	white blood cell, hemoglobin, diagnosis, prognosis	miR-329-5p/CREBRF	[22]
	circ_0121582	Down	proliferation (−)	/	miR-224/GSK3β, TET1/GSK3β/Wnt/β-catenin	[24]
CML	circ_0080145	Up	proliferation ( +)	/	sponge miR-29b	[20]
	circ_0009910	Up	proliferation ( +), autophagy ( +), apoptosis (−)	imatinib resistance, short OS	miR-34a-5p/UKL1	[47]
	circBA9.3	Up	proliferation ( +), apoptosis (−)	TKI-resistance	c-ABL1 & BCR-ABL1 level ( +)	[45]
	circ_100053	Up	/	clinical stage, BCR/ABL mutant status, short OS, imatinib resistance	/	[46]
	circ_0080145	Up	proliferation ( +), glycolysis ( +), apoptosis (−)	IM-resistance	miR-326/PPFIA1	[15]
ALL	circPVT1	Up	proliferation ( +), apoptosis (−)	/	c-Myc & Bcl-2 expression ( +)	[27]
	circAF4	Up	apoptosis (−), leukemogenesis ( +)	risk stratification	miR-128-3p/MLL-AF4	[96]
CLL	circ-CBFB	Up	progression ( +), apoptosis (−)	diagnosis, low survival time, independent predictor of prognosis	miR-607/FZD3/Wnt/β-catenin pathway	[31]
	circ-RPL15	Up	proliferation ( +)	IGHV mutation status	miR-146b-3p/RAS/RAF1/MEK/ERK pathway	[108]
	circ_0132266	Down	proliferation (−), apoptosis ( +)	/	miR-337-3p/PML	[30]
DLBCL	circ-APC	Down	proliferation (−), cell cycle (−)	Ann Arbor stage, CHOP-like and rituximab resistance, short OS, independent prognostic factor	miR-888/APC, TET1/APC, inactivate Wnt/β-catenin pathway	[33]
MCL	circCDYL	Up	proliferation ( +)	diagnosis		[34]
T-LBL	circ-LAMP1	Up	proliferation ( +), apoptosis (−)	/	miR-615-5p/DDR2	[32]
MM	circ_0007841	Up	proliferation ( +)	clinical type, cytogenetic mutation, bone destruction, R-ISS staging, DOX resistance	ABCG2 level ( +)	[49]
	circCDYL	Up	DNA synthesis ( +), apoptosis (-)	ISS and DS stage, diagnosis, short OS	miR-1180/YAP	[35]
	circ_0000190	Down	proliferation (−), apoptosis ( +), tumor growth (−)	ISS and DS stage, high risk, short PFS, OS	miR-767-5p/MAPK pathway	[37]
	circ-SMARCA5	Down	proliferation (−), apoptosis ( +)	β2-MG level, ISS stage, short PFS and OS	miR-767-5p	[126]

Note: (+) means promotion and (−) means suppression

The high expression of circHIPK3 in serum of CML patients was related to Sokal relative risk, an independent factor of CML prognosis, and shorter OS, indicating poor clinical outcome [70]. The level of circ-RPL15 was negatively correlated to the mutation state of immunoglobulin heavy chain (IGHV) gene, predicting poorer OS [108]. In DLBCL, downregulating plasma circ-APC presented diagnostic potential and was related to advanced Ann Arbor stage, low International Prognostic Index, rituximab resistance, and shorter OS [33]. The high expression of circRNA_101237 was associated with shorter OS and PFS in MM patients [109]. What is more, the expression of circRNAs in several diseases exhibited temporal specificity, which indicated that circRNAs were likely to predict clinical outcome [110]. A total of 508 circRNAs expressed dynamically throughout the treatment of all-trans retinoic acid (ATRA) in NB4 cells, and independently from the parent genes [111]. The low expression state of circ_0004277 in AML patients was diminished after chemotherapy, while the level of circ_0004277 decreased again when patients relapsed after CR, demonstrating the relationship between the increasing expression and good curative effect [112]. As consequence, the expression of circRNAs is dynamic during disease progression, which provides new aspects for therapeutic efficacy and prognosis evaluation.

The existing modalities of disease diagnosis and efficacy evaluation are invasive. Liquid biopsy, being non-invasive and repeatable, is becoming a new diagnostic tool. Accumulating evidence discovers the enrichment of circRNAs in exosomes. Exosomes protect inner circRNAs from influences of extracellular substances, making it more possible for detecting the existence of exosomal circRNAs [113]. Exosomal circRNAs act a significant part mainly in proliferation and tumor metastasis [114]. Mc-COX2, a mitochondrial genome-derived circRNA, was significantly enriched in exosomes of plasma from CLL patients, and was positively correlated with worse OS [115]. Associated with deletion 17p, t (4; 14), Durie-Salmon staging and international staging system, the level of exosomal circMYC was higher in bortezomib-resistant patients than non-resistant groups [116]. Additionally, the exosomal circ_0007841 was validated to enhance proliferation and metastasis and suppress apoptosis via activating PI3K/AKT pathway in MM cell lines [39]. Although a large number of circRNAs with biomarker value have been discovered by high-throughput sequencing, the targets and mechanisms are still unclear. The constantly emerging circRNA databases provide great convenience for target prediction and expression visualization. Here, we summarize 10 representative circRNA databases (Table 2).

**Table 2:** CircRNA databases

Name	Website address	Description	Ref.
circBase	https://circbase.org/	A public dataset of thousands of circRNAs in eukaryotic cells	[127]
circInteractome	https://circinteractome.nia.nih.gov/	A computational tool enabling the prediction and mapping of binding sites for RBPs and miRNAs on reported circRNAs	[128]
CIRCpedia V2	https://www.picb.ac.cn/rnomics/circpedia/	An updated comprehensive database containing circRNA annotations from over 180 RNA-seq datasets across six different species	[129]
circRNADb	https://reprod.njmu.edu.cn/cgi-bin/circrnadb/circRNADb.php	A comprehensive database of circular RNA molecules in humans	[130]
CSCD	https://gb.whu.edu.cn/CSCD	An integrated interactional database of cancer-specific circRNAs	[131]
exoRBase	https://www.exoRBase.org	A repository of circRNA, lncRNA and mRNA derived from RNA-seq data analyses of human blood exosomes	[132]
MiOncoCirc	https://mioncocirc.github.io/	A compendium of circular RNAs compiled from cancer clinical samples	[133]
CircAtlas 2.0	https://circatlas.biols.ac.cn/	A database of over one million of circRNAs across 6 species (human, macaca, mouse, rat, pig, chicken) and tissues	[134]
CircBank	https://www.circbank.cn/index.html	A comprehensive database of human circRNA including more than 140,000 human annotated circRNA from different source	[135]
NoncoRNA	https://www.ncdtcdb.cn:8080/NoncoRNA/	A database for experimentally supported ncRNA and drug target associations in cancer	[136]

### circRNA-related therapeutic strategies

The circRNA-miRNA-mRNA axis has become a vital mechanism in hematological tumorigenesis. As circRNAs contain multiple miRNA binding sites, targeted inhibition of circRNAs exerts more therapeutic advantages and potential than targeted inhibition of single miRNA/gene. RNA interference (RNAi) is one of the most common methods to determine the function of circRNA through loss-of-function approach. Transcripts of circRNAs could be packaged into viral vectors or oligonucleotide and then delivered to target cells to mediate their therapeutic effects [96]. Inhibiting the expression of specific circRNA could enhance the protective function of the relevant miRNAs in inhibiting oncogenes, such as XIAP, β-catenin, GSK3β and YAP [24, 31, 35, 43]. Recently, the CRISPR/Cas9-mediated genetic engineering technology provides a robust tool for circRNAs investigation. The CRISPR/Cas-assisted homologous recombination method can replace circRNA gene with a marker gene, thereby consuming circRNAs without affecting the existing gene [117]. Future investigations fueled by the well-defined guide RNA (gRNA) libraries designed for circRNA will promote the targeted therapy based on circRNA screening.

At present, a practical artificial circRNA sponge could be synthesized using simple enzymatic ligation approach. The artificial circRNA molecule is applied as an exogenous miRNA inhibitor to effectively bind and block mature miRNA, providing a promising strategy for cancer therapy [18]. Jost et al*.* engineered the artificial circRNA sponges into customized miRNA to isolate miR-122 from hepatitis C virus (HCV). In addition, circRNAs can also be used as protein sponges, and the binding sites obtained from SELEX or CLIP data can be used for many RBPs [118]. The anti-HCV circular miRNA-122 RNA sponge can be used in combination with the sequence of host factors necessary to isolate the propagation of HCV, such as hnRNP L and HuR [118]. Therefore, the artificial circRNA sponge is a promising tool in circRNA-based anti-tumor therapy, which has potential value in clinical application.

In addition, emerging evidence indicated the potential therapeutic value of tumor-related functional peptides encoded by circRNAs, especially cancer-inhibiting peptides/proteins, such as β-catenin-370aa encoded by circβ-catenin, circPPP1R12A-73aa by circPPP1R12A, and AKT3-174aa by circ-AKT3 [119]. These functional peptides can play important roles in tumorigenesis, which made them potential novel targets for drug development [119]. Due to the potential development value and clinical utility of functional peptides encoded by circRNAs, the functional peptides may be used in the research and treatment of hematological malignancies in the future.

Both the artificial circRNA and functional peptides need to be transported to the cell through an appropriate delivery system. Nanoparticles could be used to treat tumor in a variety of ways, such as intravenous injection and tail vein injection, and have become promising tools for cancer treatment. Recently, Wang et al*.* established a new plasmid delivery system, Micropoly-transfecter, which can deliver circ-1073 plasmid through intratumoral injection, thereby inhibiting tumor progression [120]. Moreover, accumulating evidence has indicated the potential value of exosomal circRNAs in clinical application [121]. Exosomes could carry circ-0051443 from normal cells to HCC cells, and inhibit the malignant biological behaviors through inducing apoptosis and cell cycle arrest [122].

CircRNAs play vital roles in the tumor microenvironment (TME) by regulating the immune surveillance and remodeling the extracellular matrix [9, 123]. CircRNA-002178 was indicated to promote the expression of PD-L1 in tumor cells through the ceRNA mechanism. Meanwhile, circRNA-002178 in tumor cells was delivered from exosomes to CD8 + T cells to achieve immune evasion of tumor cells by promoting PD-1 expression [124]. The regulation of PDL-1/PD-1 pathway by circRNA-002178 may also provide a new direction for the development of tumor-targeted drugs. Currently, the circRNA-based targeted therapy in hematological malignancies is still in its infancy. Therefore, regulation of PD-1/PD-L1 by targeting relevant circRNA may be a promising direction of future immune therapy.

## Conclusion and future perspectives

Emerging studies have revealed that the expression of circRNAs is strongly associated with tumorigenesis and prognosis of hematological malignancies. However, most of them were limited in the abnormal expression and ceRNA functions, rarely in clinical significance. Due to the convenience of circRNAs detection form peripheral blood, circRNAs may act as ideal biomarkers with the potential of clinical application. The expression of circRNAs is dynamic throughout the whole process of chemotherapy, suggesting that detecting the level of circRNAs may reflect the disease status in real time, so as to estimate the therapeutic efficacy in time. Nevertheless, such biomarkers are not specific enough for clinical appliance, which could constitute one of the focuses for future study. Current studies have confirmed that circRNAs regulate cell activity and tumor growth mainly by sponging to miRNAs and RBPs. The development of bioinformatics technology has greatly promoted the investigation of circRNA-miRNA axis. However, the specific content and mechanism of ceRNA network in hematological malignancies are still unclear. There remain a large number of circRNAs with unknown functions, and the involved mechanism is yet to be validated. Whether circRNAs participate in hematological cancers through other manners such as encoding peptides needs further investigation.

In this review, we not only summarize the contributions of circRNAs to the pathogenesis, diagnosis, chemo-resistance, and prognosis of hematological malignancies, but also put up novel biological functions and perspectives for the future clinical significances of circRNAs as therapeutic targets as well as novel treatment strategies. CircRNAs have great potential in targeted therapy due to its known regulatory functions, and the stability of circRNAs may be conducive to identify hematological malignancies by body fluids. Nevertheless, the mechanism involved in the interaction between circRNAs and hematological tumors is not fully understood yet. What’s more, although it is confirmed that circRNAs can act as potential diagnosis and prognosis biomarkers, most of the available circRNA-biomarkers are still not specific and sensitive enough to apply in clinical practice. Further studies on large-cohort prospective clinical trials will verify and promote the clinical application of circRNA biomarker candidates. Irrespective of these defects, circRNAs may still be utilized for targeted therapy in the future.

## Data Availability

Not applicable.

## Abbreviations

circRNA: Circular RNA
ceRNA: Competing endogenous RNA
ncRNA: Non-coding RNA
ecRNA: Exon circRNA
ciRNA: Circular intron RNA
EIciRNA: Exon-intron circRNA
tricRNA: TRNA intronic circular RNA
MRE: MiRNA response element
RBP: RNA-binding protein
m^6^A: N6-methyladenosine
AML: Acute myeloid leukemia
CML: Chronic myeloid leukemia
DEC: Differentially expression circRNA
ITD: Internal tandem duplication
FLT3: FMS-like tyrosine kinase-3
ALL: Acute lymphocytic leukemia
CLL: Chronic lymphocytic leukemia
T-LBL: T-cell lymphoblastic lymphoma
DLBCL: Diffuse large B-cell lymphoma
MCL: Mantle cell lymphoma
MM: Multiple myeloma
ADM: Doxorubicin
GO: Gene ontology
KEGG: Kyoto Encyclopedia of Genes and Genomes
TKI: Tyrosine kinase inhibitor
IM: Imatinib
OS: Overall survival
BTZ: Bortezomib
HK2: Hexokinase-2
GLUT: Glucose transporters
OSCC: Oral squamous cell carcinoma
PI: Propidium iodide
LDH: Lactate dehydrogenase
PCFCL: Primary cutaneous follicle center lymphoma
ROS: Reactive oxygen species
TYP: Typhaneoside
LSC: Leukemic stem cell
f-circRNA: Fusion circRNA
MLL: Mixed lineage leukemia
Ara-C: Cytarabine
ATO: Arsenic trioxide
AML-Amp: AML cases harboring MYC amplifications in the form of dmin, hsr, or ring chromosomes
EFS: Event-free survival
CR: Complete remission
ROC: Receiver operating characteristic
LFS: Leukemia-free survival
IGHV: Immunoglobulin heavy chain
NHL: Non-Hodgkin lymphoma
ATRA: All-trans retinoic acid
RNAi: RNA interference
gRNA: Guide RNA
HCV: Hepatitis C virus
TME: Tumor microenvironment
